# Osmotic gradient ektacytometry – a novel diagnostic approach for neuroacanthocytosis syndromes

**DOI:** 10.3389/fnins.2024.1406969

**Published:** 2024-07-18

**Authors:** Carolina A. Hernández, Kevin Peikert, Min Qiao, Alexis Darras, Jonathan R. A. de Wilde, Jennifer Bos, Maya Leibowitz, Ian Galea, Christian Wagner, Minke A. E. Rab, Ruth H. Walker, Andreas Hermann, Eduard J. van Beers, Richard van Wijk, Lars Kaestner

**Affiliations:** ^1^Department of Central Diagnostic Laboratory - Research, University Medical Center Utrecht, trecht University, Utrecht, Netherlands; ^2^Translational Neurodegeneration Section “Albrecht Kossel”, Department of Neurology, University Medical Center Rostock, University of Rostock, Rostock, Germany; ^3^Center for Transdisciplinary Neurosciences Rostock (CTNR), University Medical Center Rostock, Rostock, Germany; ^4^United Neuroscience Campus Lund-Rostock (UNC), Rostock, Germany; ^5^Dynamics of Fluids, Experimental Physics, Saarland University, Saarbrücken, Germany; ^6^Heoretical Medicine and Biosciences, Medical Faculty, Saarland University, Homburg, Germany; ^7^Clinical Neurosciences, Clinical and Experimental Sciences, Faculty of Medicine, University of Southampton, Southampton, United Kingdom; ^8^Physics and Materials Science Research Unit, University of Luxembourg, Esch-sur-Alzette, Luxembourg; ^9^Department of Hematology, Erasmus University Medical Center, Rotterdam, Netherlands; ^10^Department of Neurology, James J. Peters Veterans Affairs Medical Center, Bronx, NY, United States; ^11^Department of Neurology, Mount Sinai School of Medicine, New York City, NY, United States; ^12^Deutsches Zentrum für Neurodegenerative Erkrankungen (DZNE) Rostock/Greifswald, Rostock, Germany; ^13^Center for Benign Hematology, Thrombosis and Hemostasis - Van Creveldkliniek, University Medical Center Utrecht, Utrecht University, Utrecht, Netherlands

**Keywords:** VPS13A disease, XK disease, neurodegeneration, RBC deformability, ektacytometry, Osmoscan, acanthocytes

## Abstract

**Introduction:**

The unique red blood cell (RBC) properties that characterize the rare neuroacanthocytosis syndromes (NAS) have prompted the exploration of osmotic gradient ektacytometry (Osmoscan) as a diagnostic tool for these disorders. In this exploratory study, we assessed if Osmoscans can discriminate NAS from other neurodegenerative diseases.

**Methods:**

A comprehensive assessment was conducted using Osmoscan on a diverse group of patients, including healthy controls (*n* = 9), neuroacanthocytosis syndrome patients (*n* = 6, 2 VPS13A and 4 XK disease), Parkinson’s disease patients (*n* = 6), Huntington’s disease patients (n = 5), and amyotrophic lateral sclerosis patients (*n* = 4). Concurrently, we collected and analyzed RBC indices and patients’ characteristics.

**Results:**

Statistically significant changes were observed in NAS patients compared to healthy controls and other conditions, specifically in osmolality at minimal elongation index (O_min_), maximal elongation index (EI_max_), the osmolality at half maximal elongation index in the hyperosmotic part of the curve (O_hyper_), and the width of the curve close to the osmolality at maximal elongation index (O_max_-width).

**Discussion:**

This study represents an initial exploration of RBC properties from NAS patients using osmotic gradient ektacytometry. While specific parameters exhibited differences, only O_hyper_ and O_max_-width yielded 100% specificity for other neurodegenerative diseases. Moreover, unique correlations between Osmoscan parameters and RBC indices in NAS versus controls were identified, such as osmolality at maximal elongation index (O_max_) vs. mean cellular hemoglobin content (MCH) and minimal elongation index (EI_min_) vs. red blood cell distribution width (RDW). Given the limited sample size, further studies are essential to establish diagnostic guidelines based on these findings.

## 1 Introduction

Neuroacanthocytosis syndromes (NAS) comprise the neurodegenerative disorders VPS13A disease (formerly known as chorea-acanthocytosis) and XK disease (formerly known as McLeod syndrome) (Walker et al., 2023). Autosomal recessive VPS13A disease is caused by mutations in the *Vacuolar Protein Sorting 13 Homolog A* (*VPS13A*) gene and may present with progressive cognitive impairment, psychiatric symptoms, various movement disorders, muscle weakness, and epilepsy. In addition, the disease is characterized by acanthocytosis – the presence of morphologically altered red blood cells (RBCs) displaying thorn-like protrusions (Ueno et al., 2001; Rampoldi et al., 2002; Lupo et al., 2016). X-linked XK disease is caused by mutations in the *XK* gene typically leading to the absence of the Kx blood antigen. Manifestations of XK disease are very similar to VPS13A disease except for, e.g., the usually later onset and prominent cardiac involvement (Danek et al., 2001; Peikert et al., 2022a). As in VPS13A disease, acanthocytosis is a very common, but not obligatory feature (Peikert et al., 2022b). Recent studies have shown that VPS13A (bridge-like lipid transfer protein) and XK (scramblase) form a protein complex which may be the molecular basis of the phenotypical similarities in the symptoms of the related disorders (Park and Neiman, 2020; Guillén-Samander et al., 2022; Park et al., 2022; Ryoden et al., 2022).

Hence, RBCs are clearly affected in NAS (Storch et al., 2005; Siegl et al., 2013; Cluitmans et al., 2015; Peikert et al., 2022b). It is not clear whether the acanthocytes are a byproduct of the disease, directly resulting from the genetic defect, or a secondary effect. It is also unknown whether they play a role in disease progression (Franceschi et al., 2014; Adjobo-Hermans et al., 2015). It is known that RBC from NAS patients have an altered deformability (Darras et al., 2021; Rabe et al., 2021; Reichel et al., 2022; Recktenwald et al., 2022a). These properties are obviously important in the circulation of the microvasculature (Barshtein et al., 2016, 2017).

Osmotic gradient ektacytometry is another method for measuring RBC deformability, in addition to the previously mentioned microfluidic approaches (Bianchi et al., 2015; Lazarova et al., 2017; Zaninoni et al., 2018). The Laser Optical Rotational Red Cell Analyzer (Lorrca, RR Mechatronics, The Netherlands) is a device established for diagnostic parameters of RBC-related diseases (Costa et al., 2016; Kaestner and Bianchi, 2020). We tested if such an osmotic gradient ektacytometry approach could be used as a diagnostic tool for NAS. An easy method for an initial NAS screening is of utmost importance since NAS patients receive their diagnosis often very late. It is not uncommon for a correct diagnosis of NAS, which are ultra-rare diseases, to be made years or even decades after the initial symptoms appear. Obviously, the genetic confirmation is required for a definitive diagnosis (Walker and Danek, 2021), but an easy inexpensive ‘pre-test’ would be an extremely useful screening tool for both patients and clinicians.

## 2 Materials and methods

### 2.1 Patient samples

Peripheral blood was collected into EDTA tubes (Sarstedt, Germany) for healthy control samples (*n* = 9) and VPS13A disease patients (*n* = 2), XK disease patients (*n* = 4), carriers of VPS13A mutations (*n* = 3), carriers of the XK mutations (*n* = 4), Parkinson’s disease (PD) patients (*n* = 6), Huntington’s disease (HD) patients (*n* = 5) and amyotrophic lateral sclerosis (ALS) patients (*n* = 4) patients. The study was approved by the review boards of the ‘Ärztekammer des Saarlandes’, permission number 51/18, as well as of the University of Rostock (A 2019–0134), and performed in accordance with the Declaration of Helsinki. Part of the study was carried out under national (UK) research ethics committee approval 11/SC/0204 and institutional approval ERGO 41084.

### 2.2 Osmotic gradient ektacytometry

Osmoscans were performed by osmotic gradient ektacytometry on the Lorrca (RR Mechatronics, The Netherlands), according to the manufacturer’s instructions (Costa et al., 2016; Lazarova et al., 2017; Zaninoni et al., 2018). The Osmoscan was performed using a standardised final RBC dilution of 20,000 cells per μL of reagent. For the Osmoscan an osmotic gradient is created by the device by mixing two polyvinylpyrolidone (PVP) solutions with similar, physiological pH and viscosity, but different osmotic values.

For this study: Osmo LOW: 54 mOsm/kg, viscosity: 26.19 cP, Osmo HIGH: 776 mOsm/kg, viscosity: 28.76 cP was used. Data collection started at 60 mOsm/kg and continued until approximately 600 mOsm/kg. Red blood cell deformability was measured at a shear of 30 Pa, every second. The osmotic gradient gradually changes as a result of a varying mix of low and high osmotic reagent in the Couette flow system. The laser beam diffraction pattern (the A and B axis of the elliptical pattern) was measured. Deformability was expressed as the Elongation Index (EI), and calculated by the formula (A-B)/(A + B) representing the average shape change of the total RBC population under shear. The (local) minimum in elongation index (EI) in the hypo-osmotic part of the curve defines the values EI_min_ and the corresponding osmolarity as O_min_. O_min_ negatively correlates with the membrane surface-to-volume ratio of the RBCs: if the surface-to-volume ratio decreases, O_min_ increases. The (global) maximum EI of the curves define EI_max_ and as the corresponding value O_max_. If cell surface-to-volume decreases, EI_max_ decreases. The value of half EI_max_ in the hyperosmotic part of the curve defines EI_hyper_ and the corresponding osmolarity O_hyper_. An additional parameter is the area under the curve (AUC), which is correlated with the decrease in membrane deformability. Furthermore, we introduce two new parameters recently described (Wilde et al., 2023), called O_max_-width and O_min_-width, which are defined as the width of the curve at ±5% of EI_max_ and at ±5% of EI_min_. The values of O_max_-width recently showed a relation to the high intracellular viscosity of the RBC and limited ability to lose water to the environment (Wilde et al., 2023). Meanwhile O_min_-width has been correlated to RBC population volume variability, disease severity and osmotic fragility (Wilde et al., 2023).

### 2.3 Red blood cell indices

A complete RBC count including reticulocyte counts was measured by the central laboratory of the Saarland University Hospital (Flormann et al., 2022) or in the haematology department of Southampton General Hospital on an automated XN10 system (Sysmex, Japan).

### 2.4 Statistics

The various parameters of RBC and Osmoscan analyses were thoroughly examined using standard statistical methods, including ordinary one-way ANOVA and Tukey’s multiple comparison test, with a confidence interval set at 95%. The correlation heatmap was constructed using the non-parametric Spearman correlation, incorporating a two-tailed *p*-value within a 95% confidence interval. For specific correlations, linear regressions were employed. It is important to note that all of these statistical analyses were executed and visualized using GraphPad Prism. This comprehensive approach aimed to provide a robust understanding of the relationships and variations within the studied parameters, ensuring a thorough and reliable interpretation of the data.

## 3 Results

### 3.1 Patient selection and characteristics

Since both VPS13A and XK disease are very rare diseases with an estimated incidence of 1:1,000,000 and 1:10,000,000, respectively, we used the occasion of the ‘11^th^ International Meeting on Neuroacanthocytosis Syndromes’ (Kaestner, 2023), which was a joint scientific and patient meeting, to recruit two VPS13A disease patients and four XK disease patients as well as three *VPS13A* and four *XK* mutation carriers. The basic demographic characteristics of the patients are listed in Table 1. To allow a judgment for differential diagnosis, we compared the NAS patients to patients suffering from other neurodegenerative diseases, in particular, Parkinson’s disease (PD), Huntington’s disease (HD) and Amyotrophic lateral sclerosis (ALS) patients. The characteristics of these patients as well as the healthy controls are also summarized in Table 1.

**Table 1:** Patient characteristics.

		Healthy controls	Mutation carriers	NAS	HD	PD	ALS	*p*-value
Number		9	7	6	5	6	4	
Specifications			4 XK; 3 VPS13A	4 XK; 2 VPS13A	CAG repeats in the HTT gene ≧ 40	sporadic PD cases	sporadic ALS cases	
Age	Mean	54.6	64.9	51.1	65.7	69.6	63.3	0.14 (Kruskal-Wallis test)
	Min	38.4	45.4	33.6	54.1	46.2	50.1	
	Max	77.0	80.4	70.0	75.1	81.2	72.7	
	SD	14.8	12.6	13.2	9.3	12.1	11.0	
Sex	Male	4	0	6	2	4	4	0.003 (Chi-square)
	Female	5	7	0	3	2	0	

NAS, neuroacanthocytosis syndrome; HD, Huntington’s disease; PD, Parkonson’s disease; ALS, amyotrophic lateral sclerosis; CAG, codes for glutamine.

### 3.2 Osmotic gradient ektacytometry

**Figure 1:**
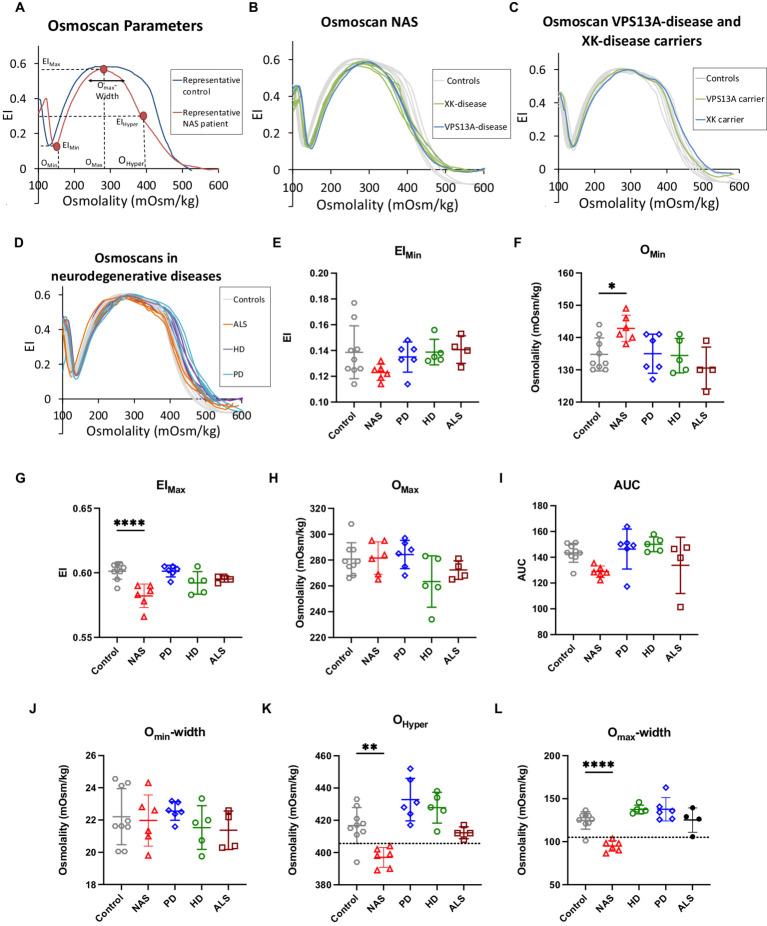
Ektacytometry data. Panel **(A)** shows representative Osmoscan curves of a NAS patient sample compared to a healthy control donor. In addition, characteristic parameters of the curve are annotated. Panel **(B)** provides the Osmoscan curves of both VPS13A disease patients and all 4 XK disease patients, showing their similarity and justifying to pool these patients in a NAS group for statistical analysis. Panel **(C)** depicts representative Osmoscan curves for both, *VPS13A* and *XK* mutation carriers showing their similarity. Panel **(D)** plots all Osmoscan curves of patients from all investigated neurodegenerative diseases except NAS to indicate the lack of difference between control curves and the neurodegenerative disorders HD, PD and ALS. In panels **(A–D)** each curve represents an individual patient or a healthy volunteer. Panels **(E–L)** show the statistical analysis of the characteristic parameters annotated in panel A. These are EI_min_, O_min_, EI_max_, O_max_, AUC, O_min_-width, O_hyper_ and O_max_-width, respectively. Plotted are the individual values with indication of the mean and the standard error of mean (SEM). Significance was checked with an ordinary one-way ANOVA test, and 1–4 stars correspond to *p*-values lower than 0.05, 0.01, 0.001, and 0.0001, respectively.

We investigated the differences in RBC deformability using the Osmoscan for patient samples from VPS13A disease, XK disease, HD, PD and ALS patients and compared it to healthy donors, in addition to carriers of VPS13A disease and XK disease. The results are summarised in Figure 1. In the graphs of Figures 1A–D, deformability vs. osmolality curves are plotted, whereas Figures 1E–L shows the statistical evaluation of particular parameters derived from the curves exemplified in Figures 1A–D.

Figure 1A depicts representative examples of Osmoscan curves from the RBCs of a healthy donor and a NAS patient. Furthermore, this panel contains annotations for the particular parameters, which are statistically evaluated. Figure 1B shows the Osmoscan curves from RBCs of both VPS13A disease patients and all four XK-disease patients, all showing the same shape characteristics justifying their pooling into one NAS group for further statistical analysis. Similarly, Figure 1C shows two VPS13A and XK disease mutation carriers measured together with nine healthy control samples, also showing the same characteristics. Figure 1D depicts representative examples from the NAS group in comparison to RBC samples from other neurodegenerative patients, in particular PD, HD and ALS.

For EI_min_ and AUC corresponding to Figures 1E–I, respectively, the curves of the NAS RBCs seem to differ from healthy control patients but this difference does not reach significance (*p* = 0.15; *p* = 0.08). In contrast, a significant difference was seen in the O_min_, EI_max_, O_hyper_ and in O_max_-width in NAS patients vs. healthy controls (Figures 1F,G,K,L). Interestingly, for O_hyper_ and O_max_-width one could find threshold values allowing discrimination between controls and NAS patients (dotted lines in Figures 1K,L, respectively) with only one false positive value from healthy controls and no false positive among the other patients. In addition, no statistically significant difference was observed for any ektacytometric parameter when comparing HD, PD and ALS vs. healthy controls.

### 3.3 Red blood cell indices and their correlation with ektacytometry parameters

**Figure 2:**
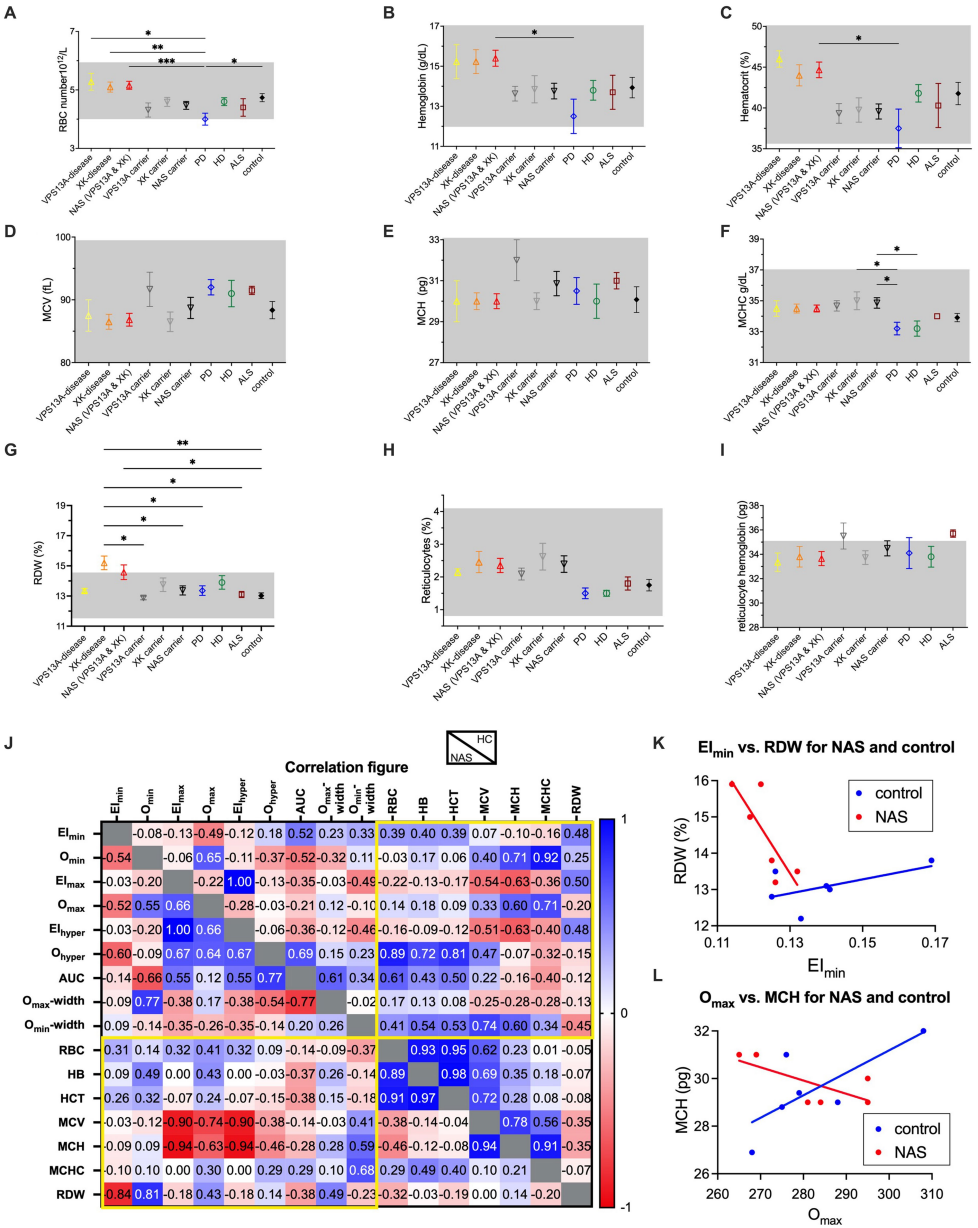
RBC indices of the different patient groups and mutation carriers and correlations with the ektacytometry parameters. Panels **(A–I)** present the RBC number per blood volume, the hemoglobin concentration of the blood, the hematocrit, the mean RBC volume (MCV), the mean RBC hemoglobin content, the mean RBC hemoglobin concentration, the RBC distribution width (RDW), the percentage of reticulocytes and the mean reticulocyte hemoglobin content, respectively. Data are based on 2 VPS13A disease patients, 4 XK disease patients, 3 *VPS13A* mutation carriers, 4 *XK* mutation carriers, 6 PD patients, 5 HD patients, 4 ALS patients and 9 controls. The NAS patients as well as the NAS mutation carriers are based on pooled respective subgroups (VPS13A and XK disease). Plotted are the mean values with the standard error of mean (SEM). Significance was checked with an Ordinary one-way ANOVA test, and 1–4 stars correspond to *p*-values lower than 0.05, 0.01, 0.001, and 0.0001, respectively. Panel **(J)** is a color-coded correlation matrix for healthy controls (HC) and pooled NAS patients of the values presented in Figures 1E–L and panels **(A–G)**. Selected correlation blots for EI_min_ vs. RDW and O_max_ vs. MCH are provided in panels K and L, respectively.

In addition, for all patients, a complete RBC count including a reticulocyte count was performed. The results are summarized in Figures 2A–I. With very few exceptions, which are the RBC distribution width (RDW) for the XK disease patients and the reticulocyte hemoglobin content for the *VPS13A* mutation carriers and the ALS patients, all measured indices were on average within the reference range (grey areas in Figures 2A–I). Differences in RBC number, hemoglobin concentration and hematocrit (Figures 2A–C, respectively) were most likely due to age and gender differences (typically, the mutation carriers were the mothers of the male patients).

Only very few parameters showed significant differences between the compared groups. Most of them were related to particular low values of the PD patients (RBC number – Figure 2A, hemoglobin concentration – Figure 2B, hematocrit – Figure 2C and mean cellular hemoglobin concentration (MCHC) – Figure 2F). However, the above-mentioned increase of RDW in XK disease patients also showed significant differences in carriers as well as ALS patients (Figure 2G). However, since the patient groups were heterogenous and not very large, such differences were likely caused by age or comorbidities rather than by the disease itself. In addition, Supplementary Table S1 provides the blood count parameters with a *p*-value for the comparison with the healthy control group.

Furthermore, we tested for correlations among all measured parameters (Figure 2J) especially the putative correlation between Osmoscan parameters and RBC indices (yellow framed areas in Figure 2J) for healthy controls and NAS patients. The correlation plot of selected parameters, namely, EI_min_ vs. RDW and O_max_ vs. MCH is provided in Figures 2K,L, respectively.

## 4 Discussion

### 4.1 Interpretation of the presented data

Deformability characteristics of RBCs have been widely used as biomarkers to determine membrane integrity and cellular viability, e.g., for disorders such as sickle cell disease (Mozar et al., 2016; Parrow et al., 2017; Connes et al., 2018; Gutierrez et al., 2021), diabetes (McMillan et al., 1978; Hanss et al., 1983; Williamson et al., 1985; Caimi and Presti, 2004) or COVID19 (Kubánková et al., 2021; Recktenwald et al., 2022b) but also in the context of RBC quality for transfusion purposes (Card et al., 1983; Barshtein et al., 2016, 2017; Lopes et al., 2023). Specifically, in NAS, membrane deformability changes have been explored along with their effect on microcirculation (Darras et al., 2021; Rabe et al., 2021; Reichel et al., 2022; Recktenwald et al., 2022a).

Although ektacytometry was already used to assess RBC deformability of NAS patients in the past (Bosman, 2018; Lazari et al., 2020), to the best of our knowledge, Osmoscans have not been explored for these neurological conditions yet. Here, we demonstrate the method’s applicability and utility for NAS by showing significant differences in several Osmoscan parameters. The best discrimination was found for O_hyper_ and O_max_-width_,_ which refers to the hydration status of the RBCs. Importantly NAS patients could be distinguished from both healthy control donors as well as other patients with neurodegenerative diseases (HD, PD and ALS).

### 4.2 Classification of the data in the diagnostic context

As outlined in the Introduction, it would be extremely useful to have an easy method for an initial NAS screening. It would be even better to have markers for the monitoring of the disease state and its progression, and for monitoring the effect of therapeutic interventions. Here we discuss to which extent osmotic gradient ektacytometry may fulfil such requirements and how it compares to other RBC-based techniques.

This is to the best of our knowledge the first approach to investigate blood samples of NAS patients by Osmoscans. Therefore, it can only be seen as an initial approach for discriminating differences in the parameters O_hyper_ – Figure 1K and O_max_-width – Figure 1L. Both O_hyper_ and O_max_-width provided only one false positive value for controls (11%) and none in the other neurodegenerative diseases (differential diagnosis). Although these initial results are very promising, sample size was small, and further studies with more patients are required to substantiate these results and inform diagnostic guidelines.

Inspired by one of the reviewers, it is suggested that the osmoscan may help differentiate between NAS patients and patients with PIEZO1 mutations [hereditary xerocytosis (HX)]. While HX patients show a left shift of their osmoscan curve (Kaestner and Bianchi, 2020), the NAS curves differ from HX in the sense that in NAS the O_min_ of the osmoscan is increased, rather than typically decreased (in HX). This results in a narrower width, which is also reflected in the significant difference in the O_max_-width parameter (Figure 1L).

Other RBC properties may be used diagnostically or as a biomarker to identify or follow NAS patients. These parameters are in particular the acanthocyte count, the erythrocyte sedimentation rate (ESR) and data derived from microfluidic approaches. Table 2 compares the different approaches including known conditions and properties of the particular techniques. The acanthocyte count is the oldest method that is purely based on the RBC shape, classifying a certain percentage of acanthocytes. This method, regardless of whether based on conventional dry blood smears or on optimized wet smears (Storch et al., 2005), proved to be challenging in practice. In patients, the number of acanthocytes can vary over time, including total absence (Malandrini et al., 1993; Sorrentino et al., 1999; Bayreuther et al., 2010). Furthermore, echinocytes can be mistaken for acanthocytes (Peikert et al., 2022b) and the method is prone to human bias (Darras et al., 2021). The situation slightly improves when 3D-imaging and shape classification based on machine learning is applied (Rabe et al., 2021; Simionato et al., 2021). Therefore the acanthocyte count as a biomarker for NAS patients can be seen as controversial.

**Table 2:** Comparison of RBC-based diagnostic measures for NAS.

Criterion	Acanthocyte count	Prolonged Erythrocyte sedimentation rate	Microfluidic assay	Ektacytometry
References	Storch et al. (2005), Darras et al. (2021), Peikert et al. (2022b)	Darras et al. (2021), Rabe et al. (2021), John et al. (2023)	Rabe et al. (2021), Recktenwald et al. (2022a), Reichel et al. (2022)	This paper
Diagnostic power	Limited (improved in automated 3D classification)	Good discrimination to controls, specificity still needs to be shown	Good discrimination to controls, specificity still needs to be shown	Discrimination at detectable limit, statistics limited to this paper
Correlation with severeness or disease state	No evidence	Unknown	Unknown	Unknown
Blood sample volume	50 μL	Typically, 1.5 mL	5 μL	200 μL
Routine devices available	Blood smear examination at different levels of automation (3D is not routine)	Several levels of automation available; parameters easy adaptable for manual devices	Yes (Erysense by Cysmic GmbH, Saarbrücken, Germany)	Yes (Lorrca by RR Mechatronics, Zwaag, The Netherlands)
Possibility of multiplexing/ throughput	Unlikely	In principle yes, but needs (software) adaptation of existing technologies	In principle yes, but needs hardware developments of existing technologies	Challenging
Implementation in clinical settings	Difficult, prone to bias, needs repeated training	Easy, though requires instrument upgrade	Medium, institution should set standard for diagnostic workup and quality control	Medium, institution should be regular user of instrument and set standard for diagnostic workup and quality control

The other methods listed in Table 2 (ESR, microfluidics and ektacytometry) have in common a more integrated read-out, which not only considers RBC shape but their deformability. Furthermore, they are recent putative biomarkers for NAS (see first line in Table 2). In this respect all of them need further investigation, studies with larger sample sizes, and therefore we are unable to favor or even recommend one of these methods as a gold-standard. Nevertheless, for each of the methods we like to highlight one up-to-date unique property that is in support for the particular approach. A significant strength of the Westergren ESR is the broad availability of this established method, including automated devices for its quantification in central haematological laboratories. It requires only a different read-out mode (a longer time and preferentially the kinetics of the sedimentation (Darras et al., 2021, 2022; Dasanna et al., 2022). The big advantage of the microfluidic approach is the small sample volume, which would very well work with samples from finger needle prick not requiring venous blood sampling (Recktenwald et al., 2022a). In this report, we present a comparison with respect to other neurodegenerative diseases for Osmoscans (currently lacking for ESR and microfluidics).

All the three methods discussed (ESR, microfluidics, and Osmoscan), are based to a large extent on RBC deformability. Therefore, all of them may develop into a diagnostic marker/biomarker. However, different deployment scenarios, such as a diagnostic screen in a large population, the follow-up of particular patients or the comparison of patients, may all require special conditions (sample volume, sample numbers, time and expenses per test) and this may determine the method of choice.

## Data availability statement

The original contributions presented in the study are included in the article/Supplementary material, further inquiries can be directed to the corresponding author.

## Ethics statement

The studies involving humans were approved by the review boards of the ‘Ärztekammer des Saarlandes’, permission number 51/18, as well as of the University of Rostock (A 2019-0134). Part of the study was carried out under national (UK) research ethics committee approval 11/SC/0204 and institutional approval ERGO 41084. The studies were conducted in accordance with the local legislation and institutional requirements. The participants provided their written informed consent to participate in this study.

## Author contributions

CH: Formal analysis, Visualization, Writing – original draft, Data curation, Investigation, Methodology. KP: Data curation, Investigation, Funding acquisition, Resources, Writing – review & editing. MQ: Data curation, Investigation, Writing – review & editing. AD: Data curation, Investigation, Writing – review & editing. JW: Writing – review & editing, Methodology. JB: Methodology, Writing – review & editing. ML: Resources, Writing – review & editing. IG: Resources, Writing – review & editing. CW: Writing – review & editing, Funding acquisition, Project administration, Supervision. MR: Writing – review & editing, Methodology. RW: Writing – review & editing, Resources. AH: Funding acquisition, Supervision, Writing – review & editing. EB: Supervision, Writing – review & editing, Methodology, Project administration. RW: Methodology, Project administration, Supervision, Writing – review & editing, Funding acquisition. LK: Funding acquisition, Project administration, Supervision, Writing – review & editing, Conceptualization, Formal analysis, Visualization, Writing – original draft.
