# Supplementary material for: Osmotic gradient ektacytometry – a novel diagnostic approach for neuroacanthocytosis syndromes
Source: Front Neurosci. 2024 Jul 18;18:1406969. doi: 10.3389/fnins.2024.1406969 (PMC11292800; doi:10.3389/fnins.2024.1406969)
Supplement: Supplementary file 1 [file Table_1.DOCX]

Supplementary Material

# Supplementary Table

**Supplementary Table S1.** Red blood cell (RBC) indices (Hb: the haemoglobin concentration of the blood, Hkt: the hematocrit, MCV: the mean RBC volume, MCH: the mean RBC haemoglobin content, MCHC: the mean RBC haemoglobin concentration, RDW: the RBC distribution width, the percentage of reticulocytes (retics)) of the different patient groups (NAS: Neuroacanthocytosis Syndrome, HD: Huntington’s disease, PD: Parkonson’s disease, ALS: Amyotrophic lateral sclerosis) compared to controls

| **index** | **Controls (n=9)** | | **NAS (n=6)** | | | **PD (n=6)** | | | **HD (n=5)** | | | **ALS (n=4)** | | |
| --- | --- | --- | --- | --- | --- | --- | --- | --- | --- | --- | --- | --- | --- | --- |
|  | **mean** | **SD** | **mean** | **SD** | **p-value*** | **mean** | **SD** | **p-value*** | **mean** | **SD** | **p-value*** | **mean** | **SD** | **p-value*** |
| **RBC number (10^12^/L)** | 4.74 | 0.42 | 5.15 | 0.33 | 0.24 | 4.05 | 0.53 | 0.013 | 4.61 | 0.33 | 0.96 | 4.40 | 0.58 | 0.55 |
| **Hb (g/dL)** | 13.9 | 1.5 | 25.4 | 0.95 | 0.26 | 12.5 | 2.1 | 0.28 | 13.8 | 1.1 | 0.99 | 13.7 | 1.7 | 0.99 |
| **Hct (%)** | 41.8 | 4.1 | 44.7 | 2.3 | 0.54 | 37.5 | 5.8 | 0.21 | 41.8 | 2.4 | 0.99 | 40.3 | 5.4 | 0.95 |
| **MCV (fL)** | 88.4 | 4.1 | 86.8 | 2.5 | 0.85 | 92.0 | 3.0 | 0.19 | 91.0 | 4.7 | 0.51 | 91.5 | 1.3 | 0.42 |
| **MCH (pg)** | 30.1 | 1.9 | 28.8 | 1.0 | 0.99 | 30.5 | 1.6 | 0.98 | 24.4 | 12.1 | 0.99 | 31.0 | 0.8 | 0.81 |
| **MCHC (g/dL)** | 33.9 | 0.8 | 34.5 | 0.5 | 0.50 | 33.2 | 1.0 | 0.33 | 33.2 | 1.1 | 0.38 | 34.0 | 0 | 0.99 |
| **RDW (%)** | 13.0 | 0.6 | 14.6 | 1.2 | 0.005 | 11.3 | 5.1 | 0.89 | 13.9 | 1.0 | 0.21 | 13.1 | 0.4 | 0.99 |
| **Retics (%)** | 1.8 | 0.3 | 2.6 | 0.5 | 0.13 | 1.5 | 0.4 | 0.75 | 1.5 | 0.2 | 0.77 | 1.8 | 0.4 | 0.99 |

* compared to the group of Controls
